# Monolithic tapered Yb-doped fiber chirped pulse amplifier delivering 126 μJ and 207 MW femtosecond laser with near diffraction-limited beam quality

**DOI:** 10.1007/s12200-023-00087-y

**Published:** 2023-10-31

**Authors:** Tao Wang, Bo Ren, Can Li, Kun Guo, Jinyong Leng, Pu Zhou

**Affiliations:** 1 College of Advanced Interdisciplinary Studies, National University of Defense Technology, Changsha, 410073 China; 2 Nanhu Laser Laboratory, National University of Defense Technology, Changsha, 410073 China; 3 Hunan Provincial Key Laboratory of High Energy Laser Technology, National University of Defense Technology, Changsha, 410073 China

**Keywords:** High-energy laser, Femtosecond laser, Tapered fiber, Fiber laser, Chirped pulse amplifier

## Abstract

In this work, a high-energy and high peak power chirped pulse amplification system with near diffraction-limited beam quality based on tapered confined-doped fiber (TCF) is experimentally demonstrated. The TCF has a core numerical aperture of 0.07 with core/cladding diameter of 35/250 µm at the thin end and 56/400 μm at the thick end. With a backward-pumping configuration, a maximum single pulse energy of 177.9 μJ at a repetition rate of 504 kHz is realized, corresponding to an average power of 89.7 W. Through partially compensating for the accumulated nonlinear phase during the amplification process via adjusting the high order dispersion of the stretching chirped fiber Bragg grating, the duration of the amplified pulse is compressed to 401 fs with a pulse energy of 126.3 μJ and a peak power of 207 MW, which to the best of our knowledge represents the highest peak power ever reported from a monolithic ultrafast fiber laser. At the highest energy, the polarization extinction ratio and the *M*^2^ factor were respectively measured to be ~ 19 dB and 1.20. In addition, the corresponding intensity noise properties as well as the short- and long-term stability were also examined, verifying a stable operation of the system. It is believed that the demonstrated laser source could find important applications in, for example, advanced manufacturing and photomedicine.

**Graphical Abstract:**

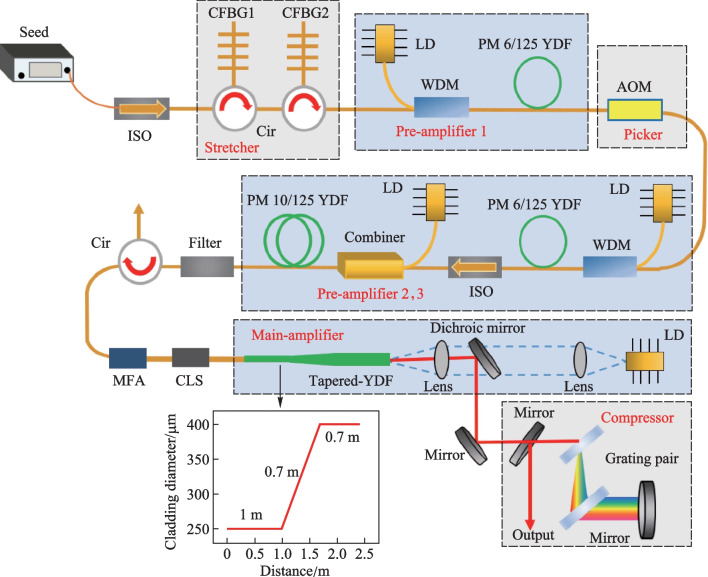

## Introduction

Ultrafast lasers with high-energy and high peak power have been widely applied in industrial fields and frontier science, such as advanced manufacturing, photomedicine, and ultrafast physics [1–4]. In general, such lasers are mainly implemented with a solid-state architecture [5, 6], which involves bulky optical systems that are free-space coupled, rendering the system fragile and cumbersome. On the contrary, fiber lasers have the advantages of flexible and compact system, high conversion efficiency, as well as excellent beam quality [7–10]. However, limited by the small mode field area and the long transmission length, the pulse energy and peak power scaling of ultrafast fiber lasers are significantly hampered by unwanted nonlinear effects [11–14].

In general, the threshold of nonlinear effects is proportional to the mode field diameter (MFD) and inversely proportional to the length of the fiber [7]. However, the fiber MFD cannot be arbitrarily increased to mitigate the nonlinear effects, as high-order mode (HOM) contents would emerge and deteriorate the output beam quality. In previous studies, large mode area (LMA) fibers with specially designed structures for suppressing HOMs were developed to boost the pulse energy and peak power of ultrafast lasers, such as photonic crystal fiber (PCF) [15, 16], large-pitch fiber (LPF) [17, 18], and chirally coupled core (3C) fiber [19, 20]. Up to now, the LPF has enabled the realization of ultrafast fiber lasers with pulse energy of 2.2 mJ [21] with peak power of several GW. However, as the specially designed structures can’t be fusion spliced to enable an all-fiber architecture, those LMA fibers generally involve bulky optical components for signal and pump collimation and coupling, leading to a sacrifice of flexibility and compactness of the laser system.

In recent years, the tapered fiber that has a longitudinally increased core/cladding diameter, has attracted much attention from the fiber laser community due to its potential to balance the nonlinear effects and the beam quality [22–26]. Essentially, the thin end of the tapered fiber can be fusion spliced with the pre-amplifier, allowing for the realization of a monolithic configuration of the high energy/power system while in the meantime maintaining a desired fundamental mode operation. In addition, the gradually enlarged MFD helps to improve the threshold of nonlinear effects, facilitating high quality amplification of ultrafast lasers. At present, the Yb-doped tapered fiber has enabled the amplification of single-frequency laser to several hundred watts with a compact all-fiber format [27, 28]. As for the ultrafast lasers, there have been lots of reports that employed active tapered fiber to directly amplify picosecond pulses to an average power of hundreds of watts and peak power of megawatts through implementing a master oscillator power amplifier (MOPA) [29–34]. Regarding the amplification of femtosecond lasers, there have also been a few demonstrations that have leveraged the tapered gain fiber. In 2015, Koptev et al. reported the amplification of picosecond laser with an Er/Yb co-doped tapered fiber, and obtained an output of 1 μJ pulses that were temporally de-chirped to 130 fs with a peak power of 2.5 MW [35]. After that, the same group utilized an Yb-doped tapered fiber to scale the energy of picosecond pulses, and obtained a compressed pulse width of 315 fs and a corresponding peak power of 22 MW at 1064 nm [36]. In 2021, a chirped pulse amplification (CPA) system was demonstrated at 1036 nm, exploiting a LMA tapered fiber, and sub-picosecond pulses with a pulse energy of 40 μJ and a peak power of 97 MW were obtained [37]. More recently, another CPA system that employed Yb-doped tapered fiber with confined doping for selectively amplifying the fundamental mode was reported to deliver hundreds of femtosecond pulses with a maximum energy of 52.4 μJ and a peak power of 132 MW, which were both limited by the accumulation of nonlinear phase that would degrade the temporal contrast and stability of the laser pulse [38].

In this study, we demonstrated a high-energy and high peak power CPA system with near diffraction-limited beam quality based on tapered confined-doped fiber (TCF) at 1030 nm. The TCF has a core numerical aperture (NA) of 0.07, and core/cladding diameters of respectively 35/250 and 56/400 μm at the thin and thick end. By temporally stretching the seed pulse to a duration of 1.7 ns with two cascading chirped fiber Bragg gratings (CFBGs), and based on the backward-pumping scheme, a maximum average power of 89.7 W and pulse energy of 177.9 μJ before pulse compression were realized. Through partially compensating for the accumulated nonlinear phase during the amplification process via adjusting the high order dispersion of one of the CFBGs, the amplified laser pulse was compressed to 401 fs with a maximum single pulse energy of 126.3 μJ by using a pair of diffraction gratings with a line density of 1739 line/mm. After excluding the pedestal components of the compressed pulse, the peak power was calculated to be 207 MW, which to the best of our knowledge represents the highest peak power that ever realized from a monolithic ultrafast fiber laser. In addition, the *M*^2^ factor was measured to be 1.20 at the highest energy, at which the long-term stable operation of the system was also verified.

## Experimental setup

**Fig. 1:**
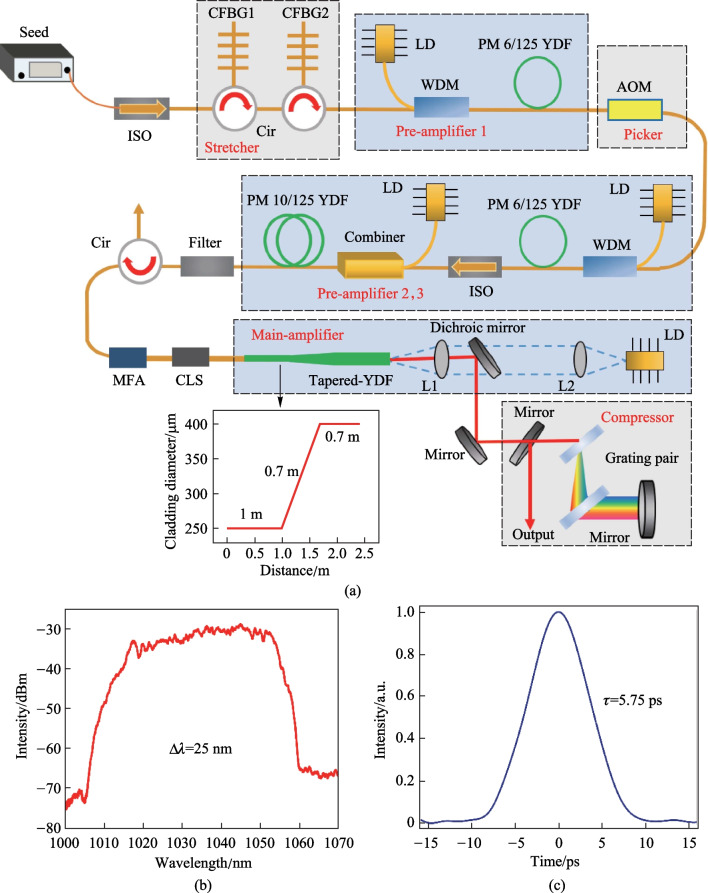
**a** Experimental setup of the monolithic TCF-CPA system: ISO: isolator; CFBG: chirped fiber Bragg grating; Cir: circulator; LD: laser diode; WDM: wavelength division multiplexer; AOM: acousto-optic modulator; MFA: mode field adaptor; PM YDF: polarization-maintaining Yb-doped fiber; CLS: clad light stripper; L1 and L2: lenses; **b** output spectrum and **c** temporal autocorrelation trace of the seed source

**Fig. 2:**
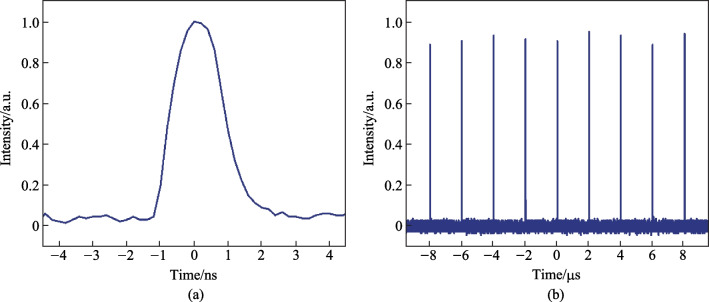
**a** Pulse temporal profile after stretching; **b** Pulse train after the pulse picker

The experimental setup of the monolithic TCF-CPA system is presented in Fig. 1a. The seed source has a maximum power of 206 mW and a repetition rate of 50.4 MHz. Its output spectrum and temporal autocorrelation trace are respectively shown in Fig. 1b and c, indicating a 3-dB spectral bandwidth of 25 nm (from 1028 to 1053 nm) and a pulse width of 5.75 ps. After passing through an isolator, the seed laser was temporally stretched by two cascaded chirped fiber Bragg gratings (CFBGs) to a duration of 1.7 ns, as shown in Fig. 2a. Both CFBG1 and CFBG2 had a central wavelength of 1030 nm, group velocity dispersion (GVD) of ~ 43 ps^2^, and 3-dB bandwidths of, respectively, 15 and 11 nm. In addition, the CFBG2 could be electronically controlled to adjust its high order dispersion via an engineered temperature profile along the CFBG. Due to the insertion loss of the pulse stretcher, the laser power was decreased to 20.9 mW, which was then boosted to 251 mW by a single-mode fiber (SMF) amplifier (pre-amplifier1), which used 3.5 m Yb-doped fiber with a core/cladding diameter of 6/125 µm and was core-pumped by a 976 nm single mode laser diode (LD). After that, the repetition rate of the laser signal was reduced to 504 kHz with a frequency reduction ratio of 100 by a pulse picker that was based on an acousto-optic modulator (AOM), which in the meantime attenuated the laser power to 1.5 mW. The corresponding pulse train is presented in Fig. 2b with a pulse interval of 1.98 μs. Subsequently, the average power was successively amplified to 130 mW by a SMF amplifier (pre-amplifier2, of which the configuration is identical to that of pre-amplifier1) and a double-clad fiber amplifier (pre-amplifier3), which used 1.1 m Yb-doped fiber with a core/cladding diameter of 10/125 μm and was cladding-pumped by a 976 nm multi-mode LD. The amplified signal was then passed through a bandpass filter with a central wavelength and bandwidth of respectively 1030 and 10 nm to filter out the amplified spontaneous emission (ASE) that was produced by the front pre-amplifiers. A circulator was then used to monitor the backward scattered light and protect the front system. A mode field adaptor (MFA), whose core/cladding diameters were respectively 10/125 and 30/250 μm at the input and output ports, was utilized to realize the transition of different mode field areas of the optical fiber. The laser signal was then injected into the main amplifier via a cladding light stripper (CLS).

The gain fiber of the main amplifier was a piece of 2.4-m-long TCF, comprised of two straight sections at the thin and thick end with core/cladding diameter of 35/250 and 56/400 μm (corresponding to a mode field area of over 1000 µm^2^), and lengths of 1 and 0.7 m, respectively. In addition, those two ends were linked by a 0.7-m-long tapered section with a taper ratio of 1.6. Moreover, the TCF had a confine-doped core that was beneficial for suppressing the HOMs and achieving high beam quality of the amplified laser. Since the TCF was drawn from a single fiber preform, its core NA of 0.07 and absorption coefficient (8 dB/m @ 976 nm) was unchanged along the fiber. To mitigate the nonlinear effects while extracting high enough laser energy, the TCF was counter-pumped at the thick end, which had a much higher mode field area and energy storage capability compared with the thin end. A multi-mode 976 nm laser diode (LD), which could deliver a maximum of 130 W average power with a NA of 0.22, was used as the pump source. The pump laser was imaged onto the end face of the TCF by a pair of lenses, L1 and L2, which had focal lengths of, respectively, 40 and 25 mm. The amplified laser was collimated by L1 and separated from the counter-propagating pump laser by using a dichroic mirror (high transmittance @ 976 nm and high reflectivity @ 1030 nm). Finally, the outputted pulse laser was temporally compressed by a pair of diffraction gratings that had central wavelengths and line densities of respectively 1030 nm and 1739 lines/mm with a Littrow angle of 63.6^◦^. It was noted that during the whole amplification process the laser signal was transmitted in an all polarization-maintaining (PM) monolithic configuration without any free-space coupling, rendering the system advantageous for stable and reliable operation.

## Results and discussion

**Fig. 3:**
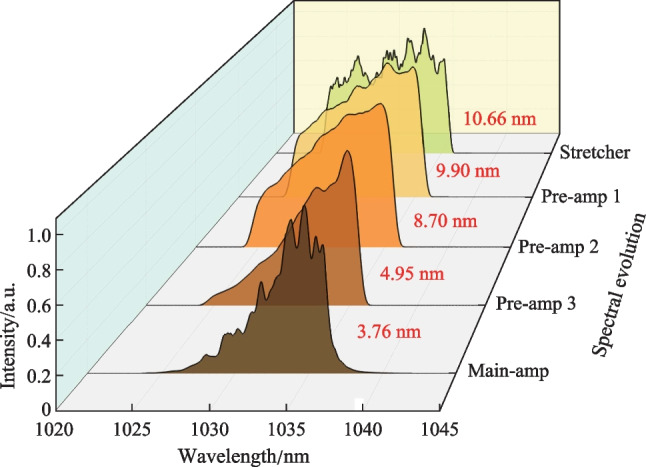
Spectral evolution of the laser signal at different amplification stages

**Fig. 4:**
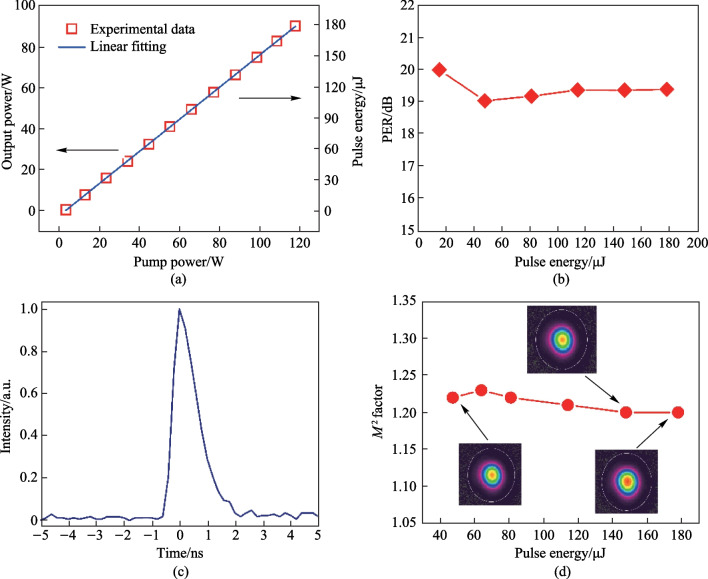
Pulse characteristics of the main-amplifier: **a** output power and single pulse energy versus the pump power; **b** PER at different pulse energies; **c** temporal pulse waveform at the highest output power; **d** *M*^2^ factor at different single pulse energies

Figure 3 presents the spectral evolution of the laser signal at different amplification stages. Owing to the limitation of the bandwidth of CFBGs, the 3 dB bandwidth of the laser spectrum after the stretcher was measured to be 10.66 nm, which was then gradually narrowed along the amplification chain to a minimum of 3.76 nm at the highest output power due to the gain-narrowing effect. Figure 4a shows the variation of output power and pulse energy evolution with increasing pump power. It can be seen that the output power/energy was linearly enhanced to a maximum of 89.7 W/177.9 μJ at pump power of 118 W, corresponding to an optical-to-optical conversion efficiency of 76%. Further scaling of the output power was limited by the onset of stimulated Raman scattering (SRS) effect. The polarization extinction ratio (PER) at different pulse energies was also characterized by using a half-wave plate and a polarized beam splitter (PBS), and the results are shown in Fig. 4b. With further increase of the single pulse energy, the PER remained larger than 19 dB and relatively stable. At the highest output power, the pulse temporal waveform was also recorded by a photodetector (bandwidth: 5 GHz) and an oscilloscope (bandwidth: 1 GHz, sampling speed: 5 GS/s) and shown in Fig. 4c, which demonstrates a pulse width of 996 ps. Owing to the gain saturation effect, the leading edge of the pulse extracted more gain, leading to a steeper leading edge of the pulse compared with the trailing edge. However, this effect could be well circumvented through pre-shaping the pulse waveform, i.e., by intentionally suppressing the long wavelength components of the seed laser via an engineered spectral filter. Figure 4d shows the evolution of the beam quality (an average between the horizontal and vertical directions) during the energy scaling progress. It can be observed that the *M* ^2^ factor keeps at around 1.20 and changes little with the increase of the pulse energy, indicating a near diffraction-limited beam quality.

**Fig. 5:**
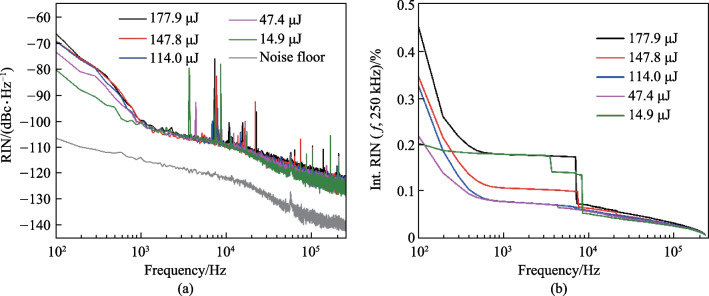
Intensity noise properties of the amplified laser at different single pulse energies: **a** RIN spectra and **b** corresponding integrated RIN

In addition, the relative intensity noise (RIN) of the amplified laser was characterized in the frequency range of 100 Hz–250 kHz with a low noise photodetector, a low pass filter with cut-off frequency of 500 kHz, and an electrical spectrum analyzer (ESA). The measured RIN spectra and corresponding integrated RIN at different single pulse energies are respectively presented in Fig. 5a and b. In general, the dominating noise sources in the low frequency range were mainly attributed to the technical noises such as environmental disturbances and pump power fluctuations. As such, the RIN spectra demonstrated a monotonously decreasing trend in the examined frequency range. As demonstrated in Fig. 5a, the amplitude of the RIN spectrum at 10–250 kHz became slightly elevated with increase of the pulse energy and reached − 124 dBc/Hz@250 kHz at the highest pulse energy of 177.9 μJ. Similar behavior was observed at frequencies lower than 1 kHz, and the possible reason is that the accumulation of nonlinear phase induced degradation of the pulse quality. Correspondingly, the integrated RIN at the examined frequency range was increased from 0.20% to 0.46%, as shown in Fig. 5b. Moreover, the noise spikes presenting on the noise spectra were attributed to the electronical pick-up noise that brought in by the driving electronics of the pump laser [39, 40].

**Fig. 6:**
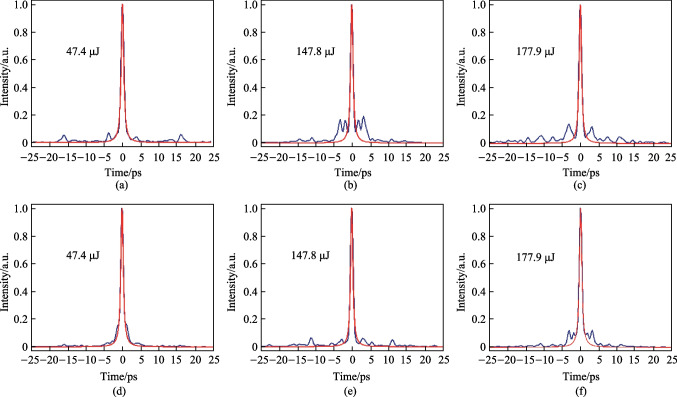
Temporal autocorrelation trace of the compressed pulse at selected single pulse energies of 47.4 μJ (**a**, **d**), 147.8 μJ (**b**, **e**), and 177.9 μJ (**c**, **f**). The upper and lower rows respectively represent the case without and with compensation of the nonlinear effects. The blue and red curves are respectively the measured autocorrelation trace and fitted curve

**Fig. 7:**
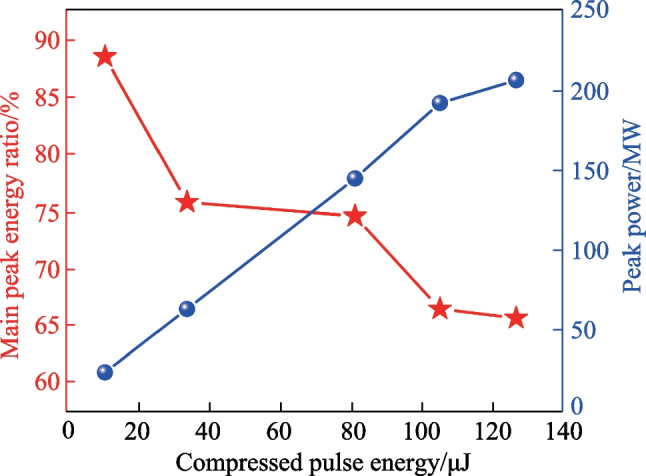
Main peak energy ratio and peak power of compressed pulses at different single pulse energies

Regarding the compressed pulse, its temporal autocorrelation trace was measured at selected energies and is shown in Fig. 6, in which the upper and lower rows respectively represent the case without and with compensation of the nonlinear effects through finely adjusting the high-order dispersion (HOD) of CFBG2. Apparently, owing to the uncompensated HOD and nonlinear effects, the quality of the compressed pulse was deteriorated by emergence of the pedestal and satellite peaks with increasing of the pulse energy. Fortunately, by finely adjusting the HOD of CFBG2, the compressed pulse quality was considerably improved, as reflected by the shrinking of the pedestal and the increase of the ratio of pulse energy in the main peak. Nevertheless, the proportion of the main peak energy relative to the whole pulse (calculated by dividing the integration of the fitted curve into that of the measured autocorrelation trace) still gradually decreased with the scaling of the pulse energy (shown in Fig. 7); solely adjusting the HOD could not be leveraged to completely compensate for the nonlinear effects, especially at higher operation energy. Also shown in Fig. 7 is the calculated peak power, which first demonstrates a linearly increasing trend and then a slight saturation phenomenon, indicating that extra methods such as pulse shaping are required to achieve higher peak power [41]. With a compression efficiency of 71%, the highest compressed pulse energy was 126.3 μJ with a pulse width of 401 fs (Lorentz fitting) and a main peak energy proportion of 65.7%, corresponding to a maximum peak power of 207 MW.

**Fig. 8:**
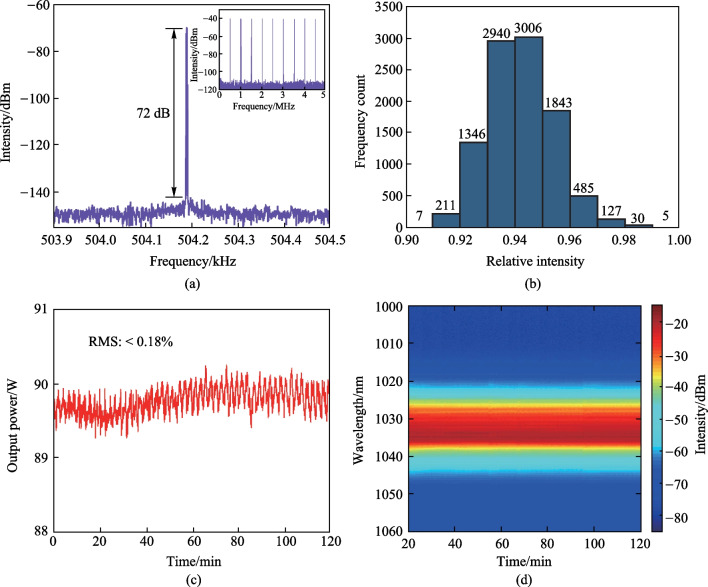
Stability characterization of the fiber amplifier at the highest operation energy: **a** RF spectrum at the fundamental frequency; inset, RF spectrum in the frequency range of 5 MHz; **b** statistical histogram of the amplitude of 10,000 pulses with respect to the maximum; **c** average power and **d** spectrum evolutions recorded over 2 h

To verify the stable operation of the fiber amplifier, the radio frequency (RF) spectrum at the fundamental frequency with a resolution bandwidth (RBW) of 1 Hz at the highest energy was measured and is shown in Fig. 8a, which demonstrates a signal-to-noise of 72 dB. The inset presents the RF spectrum in the frequency range of 5 MHz with a RBW of 1 kHz, indicating that the amplified laser had a good temporal stability. Besides, the amplitude of 10,000 pulses was counted and normalized to the maximum, to generate the corresponding statistical histogram in Fig. 8b, showing a normal distribution of the pulse amplitude fluctuation. In addition, the amplitude fluctuation (standard deviation to the mean) was calculated to be 1.3% over the recorded pulses. Figure 8c depicts the average power evolution recorded every second for 2 h, demonstrating a power fluctuation of less than 0.18% (root mean square, RMS). The slight fringes presenting on the recorded curve were attributed to the temperature drifting induced pump power fluctuations. The corresponding spectral evolution was recorded every 15 s and is shown in Fig. 8d, in which the spectrum profile hardly changed over the monitored period.

## Conclusion

In conclusion, we report a high-energy and high peak power monolithic CPA system with near diffraction-limited beam quality based on a TCF, which has the core NA of 0.07 with core/cladding diameter of 35/250 µm at the thin end and 56/400 μm at the thick end. Through implementing a backward-pumping scheme, the maximum pulse energy of 126.3 μJ was obtained after pulse compression at 504 kHz. Meanwhile, with adjustment of the high order dispersion of one of the CFBGs, the amplified pulse duration was shortened to 401 fs by a pair of diffraction gratings. Excluding the energy in the pulse pedestal, the peak power of the compressed pulse was estimated to be 207 MW, which represents the highest peak power generated from a monolithic fiber laser ever reported, to our knowledge. At the highest energy, the PER and the *M*^2^ factor were respectively measured to be ~ 19 dB and 1.20. In addition, the corresponding intensity noise properties as well as the short- and long-term stability were also examined, verifying a stable operation of the system.

## Data Availability

The data that support the findings of this study are available from the corresponding author, upon reasonable request.
